# Ceratinadin G, a new psammaplysin derivative possessing a cyano group from a sponge of the genus *Pseudoceratina*

**DOI:** 10.3762/bjoc.20.267

**Published:** 2024-12-09

**Authors:** Shin-ichiro Kurimoto, Kouta Inoue, Taito Ohno, Takaaki Kubota

**Affiliations:** 1 Faculty of Medicine, Dentistry, and Pharmaceutical Sciences, Okayama University, 1-1-1, Tsushima-naka, Kita-ku, Okayama 700-8530, Japan; 2 Showa Pharmaceutical University, 3-3165 Higashi-Tamagawagakuen, Machida, Tokyo 194-8543, Japan

**Keywords:** ceratinadin, cytotoxicity, marine sponge, psammaplysin, *Pseudoceratina* sp.

## Abstract

A new psammaplysin derivative, ceratinadin G (**1**), was obtained from the Okinawan marine sponge *Pseudoceratina* sp., and the gross structure was clarified through spectroscopic and spectrometric analyses. The absolute configuration of compound **1** was established by comparing its NMR and ECD data with those of the known psammaplysin derivative, psammaplysin F (**2**). Ceratinadin G (**1**) is a rare nitrile containing a cyano group as aminoacetonitrile, and is the first psammaplysin derivative possessing a cyano group. In vitro assays indicated that compound **1** displayed moderate cytotoxicity against L1210 murine leukemia cells and KB epidermoid carcinoma cells.

## Introduction

**Figure 1:**
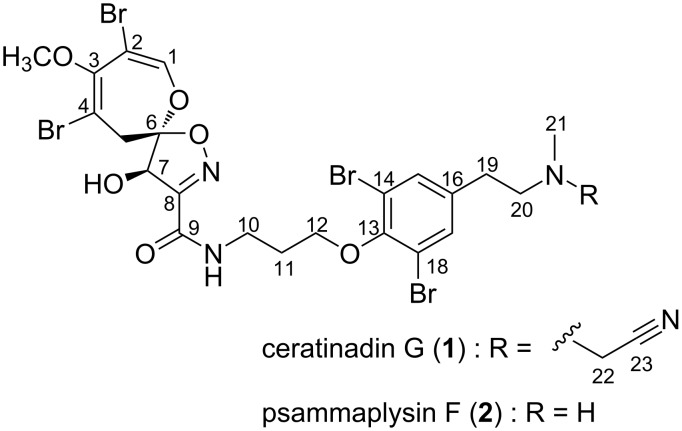
Structures of ceratinadin G (**1**) and psammaplysin F (**2**).

Marine sponges are widely recognized as a rich source of unique bioactive natural products. For instance, marine sponges belonging to the order Verongiida are known to contain a diverse array of bromotyrosine alkaloids with a broad spectrum of biological activities [1]. To date, approximately 500 bromotyrosine alkaloids have been isolated from sponges, with those featuring the 8,10-dibromo-9-methoxy-1,6-dioxa-2-azaspiro[4.6]undeca-2,7,9-trien-4-ol moiety classified as psammaplysin derivatives. About 50 psammaplysin derivatives have been identified so far [2–3]. Among bromotyrosine alkaloids, the psammaplysin derivatives are particularly intriguing due to their structural complexity and biological activities. Psammaplysin derivatives exhibit a range of bioactivities, including antibacterial, anticancer, antimalarial, and antiviral effects. Since the discovery of the first psammaplysin derivative, psammaplysin A [4–5], these alkaloids have been recognized as challenging targets for total synthesis. The absolute configuration of psammaplysin A remained ambiguous for approximately 30 years but was determined in 2015 by Kurtán, Garson, and co-workers through a comparison of experimental and calculated electronic circular dichroism data, as well as a method employing Trost's chiral anisotropic reagents [6]. More recently, the first asymmetric total synthesis of psammaplysin A was accomplished by Smith and Morrow, and the absolute configuration of compound **1** was also confirmed through organic synthesis [7]. In our ongoing research focused on uncovering new bioactive secondary metabolites from Okinawan marine sponges, we have identified various bioactive bromotyrosine alkaloids [8]. Previously, we isolated two psammaplysin derivatives, ceratinadins E and F, containing two or three 11-*N*-methylmoloka’iamine units, from the Okinawan marine sponge *Pseudoceratina* sp. [9]. Further investigation of this sponge has led to the discovery of an additional psammaplysin derivative, ceratinadin G (**1**) (Figure 1). This paper details the isolation, structural elucidation, and biological activity of compound **1**.

## Results and Discussion

The marine sponge *Pseudoceratina* sp., collected in Okinawa, Japan, was initially extracted with MeOH, after which the extract was partitioned between EtOAc and H_2_O. The EtOAc-soluble fraction was subjected to silica gel column chromatography. A detailed analysis of the ^1^H NMR spectrum of the fraction eluted after the one containing ceratinadins E and F revealed signals corresponding to psammaplysin F (**2**), as well as additional signals not matching those of any known psammaplysin derivatives. Further separation of this fraction using C_18_ HPLC led to the isolation of ceratinadin G (**1**, 0.4 mg, 3.2 × 10^−4^%) along with psammaplysin F (**2**) [10–12].

Ceratinadin G (**1**) was isolated as an optically active, colorless, amorphous solid. ESIMS analysis of compound **1** revealed pseudomolecular ion peaks at *m/z* 805, 807, 809, 811, and 813 (1:4:6:4:1 [M + Na]^+^), which is indicative of the presence of four bromine atoms. The molecular formula of compound **1**, determined by HRESIMS, was identified as C_24_H_26_Br_4_N_4_O_6_. The existence of a substituted benzenoid chromophore was suggested by the UV absorption maximum at 258 nm. The presence of hydroxy and/or amino groups and a carbonyl group was indicated by IR absorptions at 3337 cm^−1^ and 1671 cm^−1^, respectively. The analysis of the HSQC spectrum, along with the ^1^H and ^13^C NMR data, revealed that compound **1** contains eleven non-hydrogen-bearing carbons, three sp^2^ methines, one sp^3^ methine, seven sp^3^ methylenes, and two methyls (Table 1).

**Table 1:** ^1^H and ^13^C NMR data of ceratinadin G (**1**) in methanol-*d*_4_.

Position	δ_H_^a^	multi (*J* in Hz)	δ_C_^b^	multi

1	7.18	s	147.6	d
2	–		105.2	s
3	–		150.7	s
4	–		105.3	s
5a	3.43	d (16.1)	39.0	t
5b	3.11	d (16.1)		
6	–		121.7	s
7	5.02	s	81.2	d
8	–		159.6	s
9	–		161.5	s
10	3.66^c^	td (7.1, 1.1)	38.8	t
11	2.17^c^	tt (7.1, 6.0)	31.4	t
12	4.10^c^	t (6.0)	72.9	t
13	–		153.5	s
14	–		119.7	s
15	7.52^c^	s	135.0	d
16	–		141.3	s
17	7.52^c^	s	135.0	d
18	–		119.7	s
19	2.78^c^	t (6.8)	33.9	t
20	2.73^c^	t (6.8)	58.7	t
21	2.43^d^	s	42.8	q
22	3.73^c^	s	46.3	t
23	–		116.9	s
3-OCH_3_	3.69^d^	s	60.1	q

^a^600 MHz; ^b^150 MHz; ^c^2H; ^d^3H.

**Figure 2:**
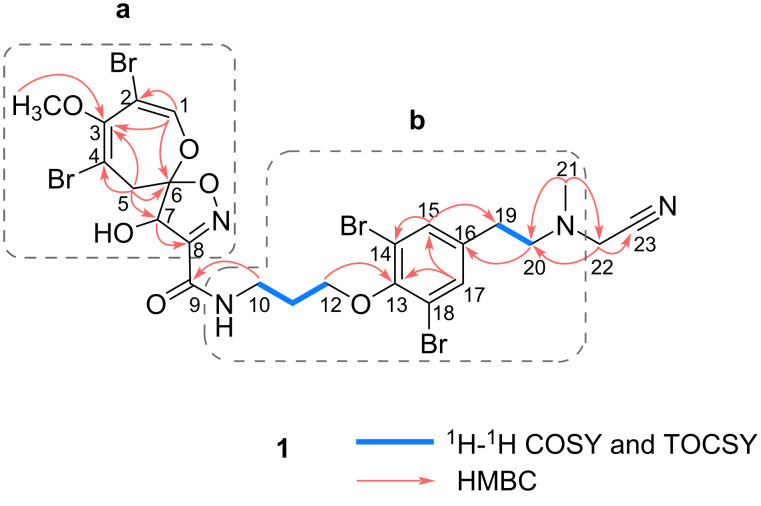
Selected 2D NMR correlations for ceratinadin G (**1**).

The existence of the 8,10-dibromo-9-methoxy-1,6-dioxa-2-azaspiro[4.6]undeca-2,7,9-trien-4-ol unit and the 11-*N*-methylmoloka'iamine unit (partial structures **a** and **b**, respectively, in Figure 2), which were characteristic of psammaplysins, in ceratinadin G (**1**) was suggested by comparison of its ^1^H and ^13^C NMR data with those of known psammaplysin derivatives such as psammaplysins A and F (**2**) [4–6,10–12]. HMBC correlations (H-1/C-2, H-1/C-3, H-1/C-6, H_2_-5/C-3, H_2_-5/C-4, H_2_-5/C-6, H-5a/C-7, H-7/C-8, and 3-OCH_3_/C-3) supported the presence of the 8,10-dibromo-9-methoxy-1,6-dioxa-2-azaspiro[4.6]undeca-2,7,9-trien-4-ol unit (partial structure **a** in Figure 2). While the existence of the 11-*N*-methylmoloka'iamine unit (partial structure **b** in Figure 2) was confirmed by the ^1^H-^1^H COSY and TOCSY correlations (C-10–C-12 and C-19–C-20), and HMBC correlations (H_2_-12/C-13, H-15/C-14 (H-17/C-18), H-15/C-19 (H-17/C-19), H-17/C-13 (H-15/C-13), H-17/C-15 (H-15/C-17), and H_2_-20/C-16). HMBC correlations between the *N*-methyl protons H_3_-21 (δ_H_ 2.43) and methylene carbon C-22 (δ_C_ 46.3), and between H_2_-22 (δ_H_ 3.73) and *N*-methylene carbon C-20 (δ_C_ 58.7), indicated that C-22 was connected to 20-N. Additionally, an HMBC correlation was observed between H_2_-22 and the non-hydrogen-bearing carbon C-23. Considering the chemical shift of C-23 (δ_C_ 116.9) and the molecular formula of compound **1**, it was inferred that a cyano group is attached to C-22. The ^13^C NMR chemical shifts of C-22 and C-23 closely matched those of the corresponding carbons in known synthetic compounds with an aminoacetonitrile moiety, further supporting the presence of a cyano group in compound **1** [13–15]. In the IR spectrum of **1**, an absorption attributed to the stretching vibration of the C≡N bond was observed at 2234 cm^−1^, although its intensity was very weak (Figure S8 in Supporting Information File 1). It is known that when an atom with an electron-withdrawing inductive effect is attached to the carbon bearing the cyano group, the intensity of the absorption derived from the cyano group in the IR spectrum decreases significantly. This phenomenon has been reported, particularly in compounds where halogen or oxygen atoms are bonded to the carbon bearing the cyano group [16]. A similar effect is considered to occur when a nitrogen atom, which has a greater electronegativity than carbon, is attached. In fact, it has been reported that in aminoacetonitrile derivatives, the absorption due to the cyano group in the IR spectrum is either very weak or not observed [15]. Despite the absence of an HMBC correlation directly indicating a connection between C-8 and C-9, the HMBC correlation between the *N*-methylene protons H_2_-10 (δ_H_ 3.66) and the carbonyl carbon C-9 (δ_C_ 161.5), along with the molecular formula of compound **1** by process of elimination, suggested that C-8 and 9-N were linked via a carbonyl group at C-9. Therefore, the gross structure of **1** was elucidated.

**Figure 3:**
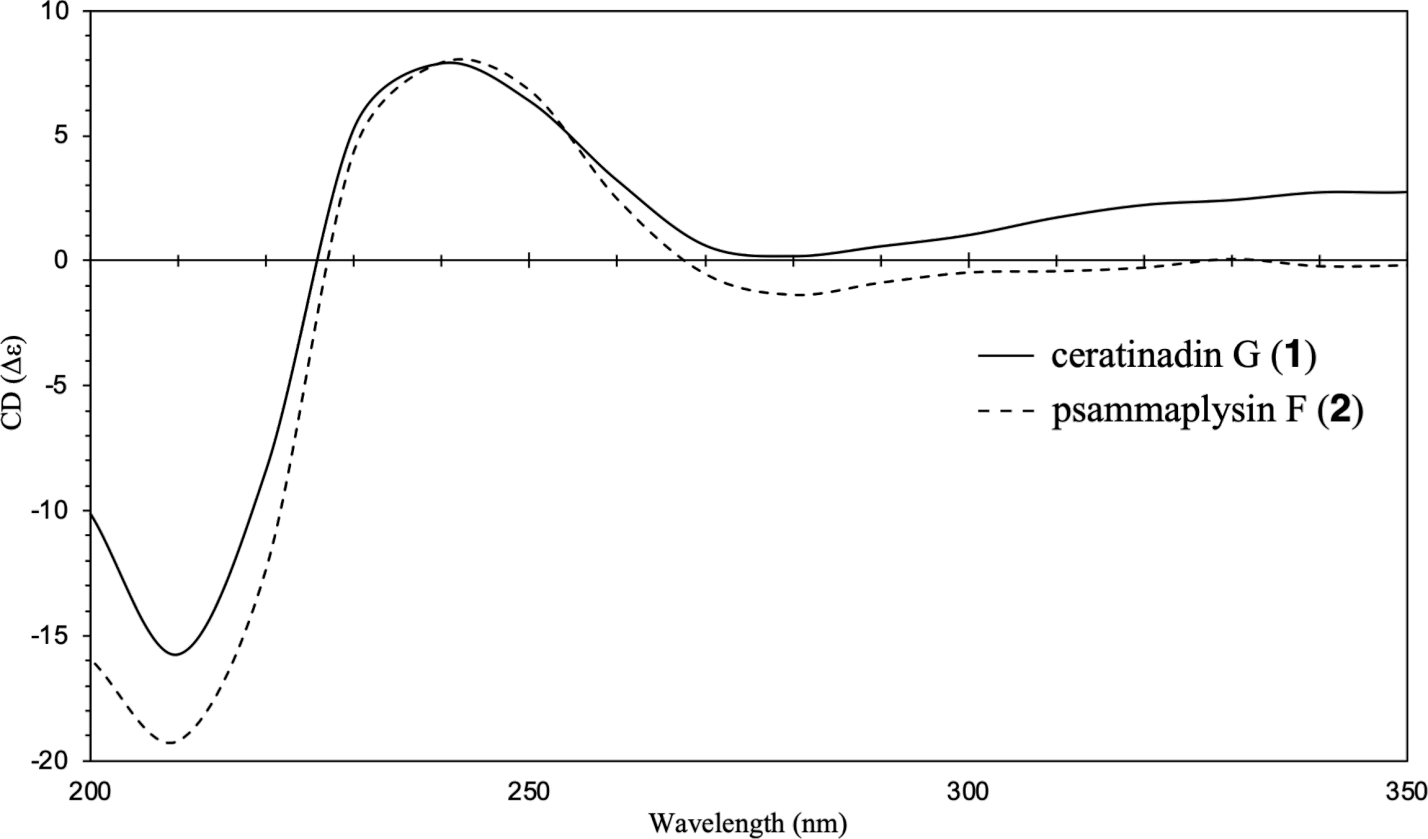
ECD spectra of ceratinadin G (**1**) and psammaplysin F (**2**) in MeOH.

The absolute configuration of ceratinadin G (**1**) was assigned by comparing its NMR and ECD data with those of psammaplysin F (**2**), whose absolute configuration has already been established [10–12]. The NMR data of the 8,10-dibromo-9-methoxy-1,6-dioxa-2-azaspiro[4.6]undeca-2,7,9-trien-4-ol moiety of **1** and the ECD spectrum pattern of **1** matched those of compound **2** (Figure 3). Consequently, the absolute configuration of **1** was assigned as 6*R* and 7*R*, identical to that of **2**.

The in vitro cytotoxicity of ceratinadin G (**1**) was evaluated against L1210 murine leukemia cells and KB epidermoid carcinoma cells. Ceratinadin G (**1**) showed moderate cytotoxic activity against L1210 cells (IC_50_ = 4.7 μM) and KB cells (IC_50_ = 15.1 μM).

## Conclusion

Ceratinadin G (**1**) is the first psammaplysin derivative identified to contain a cyano group. Bromotyrosine alkaloids with cyano groups have been discovered in marine sponges and are classified into two categories. One type has a cyano group as part of a phenylacetonitrile structure [17–24], while the other type contains a cyano group as cyanoformamide [25–28]. It is known that natural nitrile compounds are biosynthesized through various mechanisms [29]. Rinehart and co-workers demonstrated that 2-(3,5-dibromo-4-hydroxyphenyl)acetonitrile is biosynthesized from ʟ-tyrosine via 3,5-dibromo-ʟ-tyrosine, based on experiments using ^14^C- and ^15^N-labeled ʟ-phenylalanine [19]. Therefore, the cyano group in bromotyrosine alkaloids containing the phenylacetonitrile moiety is derived from the α-carbon and amino group of ʟ-tyrosine. On the other hand, the biosynthesis of nitrile with a cyanoformamide moiety remains unclear. Ceratinadin G (**1**) represents a rare nitrile that contains a cyano group as aminoacetonitrile. The biosynthesis of the 8,10-dibromo-9-methoxy-1,6-dioxa-2-azaspiro[4.6]undeca-2,7,9-trien-4-ol scaffold has been proposed by Scheuer, Clardy and co-workers [5], but how the cyano group in **1** is biosynthesized remains unknown and is of significant interest.

## Experimental

### General experimental procedures

Optical rotations were measured using a JASCO P-2200 polarimeter. UV spectra were obtained with a JASCO Ubest-55 spectrophotometer. IR spectra were recorded on a JASCO FT/IR-420 spectrophotometer. The ECD spectra were measured using a JASCO J-1500 spectropolarimeter. ^1^H and ^13^C NMR spectra were acquired on a Bruker Avance II 600 MHz NMR spectrometer equipped with a cryoplatform, using 3.0 mm micro cells (Shigemi Co., Ltd.) for CD_3_OD. The ^1^H NMR chemical shift of residual CD_2_HOD in CD_3_OD at 3.35 ppm and the ^13^C NMR chemical shift of CD_3_OD at 49.8 ppm were used as internal references. Mass spectra were acquired on a JEOL JMS-T100LP spectrometer. Flash column chromatography was performed using a Biotage Isolera flash purification system.

### Extraction and isolation

The EtOAc-soluble material (2.45 g) of the methanol extract (38.06 g) from the sponge *Pseudoceratina* sp. (order Verongida; family Aplysinellidae), collected in Okinawa, Japan (0.4 kg, wet weight), was obtained following the method described in [8]. In a manner similar to [8], a portion of the EtOAc-soluble material (1.45 g) was fractionated by silica gel column chromatography [silica gel 60N (spherical, neutral, 40–50 µm), Kanto Chemical Co., Inc.; 38 × 350 mm; eluent CHCl_3_/MeOH 100:0 to 0:100], yielding 18 fractions (Fr. 1–18). A portion (17.0 mg) of the fraction Fr. 14 (32.5 mg) was further separated by C_18_ HPLC (COSMOSIL 5C18-AR-II, 10 × 250 mm, Nacalai tesque Inc.; eluent MeCN/H_2_O/TFA 45:55:0.1; flow rate 2.5 mL/min; UV detection at 254 nm), yielding psammaplysin F (**2**, *t*_R_ 14 min, 6.9 mg, 0.0056% wet weight) and ceratinadin G (**1**, *t*_R_ 56 min, 0.4 mg, 0.00032% wet weight).

Ceratinadin G (**1**): colorless amorphous solid; [α]_D_^27^ −71.0 (*c* 0.02, MeOH); UV (MeOH) λ_max_, nm: 207 (ε 28138) and 258 (ε 10233); IR (film/KBr) ν_max_: 3337, 2935, 2878, 2849, 2234 (weak), 1671, 1624, 1595, 1542, 1457, 1257, 1199, 1145, 1119, 1046, 954, 898, 738 cm^−1^; ECD (MeOH) λ_max_, nm: 211 (Δε −15.84), 239 (Δε 7.99), 281 (Δε 0.07); ^1^H and ^13^C NMR data (Table 1); HRESIMS (*m/z*): [M + Na]^+^ calcd for C_24_H_26_^79^Br_2_^81^Br_2_N_4_O_6_Na, 808.84426; found, 808.84502.

### Cytotoxicity assay

L1210 murine leukemia cells were cultured in RPMI-1640 supplemented with 10% FBS, and KB epidermoid carcinoma cells were cultured in DMEM supplemented with 10% FBS. All cells were incubated at 37 °C in a humidified atmosphere of 5% CO_2_ and 95% air. Cells were seeded at a density 2 × 10^3^ cells/well (198 μL/well) in 96-well plates, and test samples dissolved in DMSO (2 μL) were added to each well. The cells were then incubated for 72 hours. Cell viability was assessed using a WST-8 [2-(2-methoxy-4-nitrophenyl)-3-(4-nitrophenyl)-5-(2,4-disulfophenyl)-2*H*-tetrazolium monosodium salt] colorimetric assay. WST-8 solution (10 μL) was added to each well, and after an additional 4 hours of incubation, absorbance at 450 nm was measured using an Infinite M200 microplate reader (TECAN). Paclitaxel and vincristine were used as positive controls.

## Supporting Information

File 1 ^1^H NMR, ^13^C NMR, ^1^H-^1^H COSY, TOCSY, HSQC, HMBC, NOESY, and IR spectra of caratinadin G (**1**).

## Data Availability

All data that supports the findings of this study is available in the published article and/or the supporting information of this article.
