# Supplementary material for: Ceratinadin G, a new psammaplysin derivative possessing a cyano group from a sponge of the genus Pseudoceratina
Source: Beilstein J Org Chem. 2024 Dec 9;20:3215–20. doi: 10.3762/bjoc.20.267 (PMC11650486; doi:10.3762/bjoc.20.267)
Supplement: File 1 — 1H NMR, 13C NMR, 1H-1H COSY, TOCSY, HSQC, HMBC, NOESY, and IR spectra of caratinadin G (1). [file Beilstein_J_Org_Chem-20-3215-s001.pdf]

BEILSTEIN JOURNAL OF ORGANIC CHEMISTRY

Supporting Information

for

Ceratinadin G, a new psammaplysin derivative possessing a cyano group from a sponge of the genus *Pseudoceratina*

Shin-ichiro Kurimoto, Kouta Inoue, Taito Ohno and Takaaki Kubota

Beilstein J. Org. Chem. **2024,** *20,* 3215–3220. doi:10.3762/bjoc.20.267

^{1}H NMR, ^{13}C NMR, ^{1}H-^{1}H COSY, TOCSY, HSQC, HMBC, NOESY, and IR spectra of caratinadin G (1)

License and Terms: This is a supporting information file under the terms of the Creative Commons Attribution License (https://creativecommons.org/licenses/by/4.0). Please note that the reuse, redistribution and reproduction in particular requires that the author(s) and source are credited and that individual graphics may be subject to special legal provisions.
The license is subject to the *Beilstein Journal of Organic Chemistry* terms and conditions: (https://www.beilstein-journals.org/bjoc/terms)

Table of Contents:

Figure S1. ^{1}H NMR spectrum of ceratinadin G (**1**) in CD_3OD (600 MHz).
Figure S2. ^{13}C NMR spectrum of ceratinadin G (**1**) in CD_3OD (150 MHz).
Figure S3. ^{1}H-^{1}H COSY spectrum of ceratinadin G (**1**) in CD_3OD (600 MHz).
Figure S4. TOCSY spectrum of ceratinadin G (**1**) in CD_3OD (600 MHz).
Figure S5. HSQC spectrum of ceratinadin G (**1**) in CD_3OD (600 MHz).
Figure S6. HMBC spectrum of ceratinadin G (**1**) in CD_3OD (600 MHz).
Figure S7. NOESY spectrum of ceratinadin G (**1**) in CD_3OD (600 MHz).
Figure S8. IR spectrum of ceratinadin G (**1**).

Figure S1: ^{1}H NMR spectrum of ceratinadin G (**1**) in CD_3OD (600 MHz).

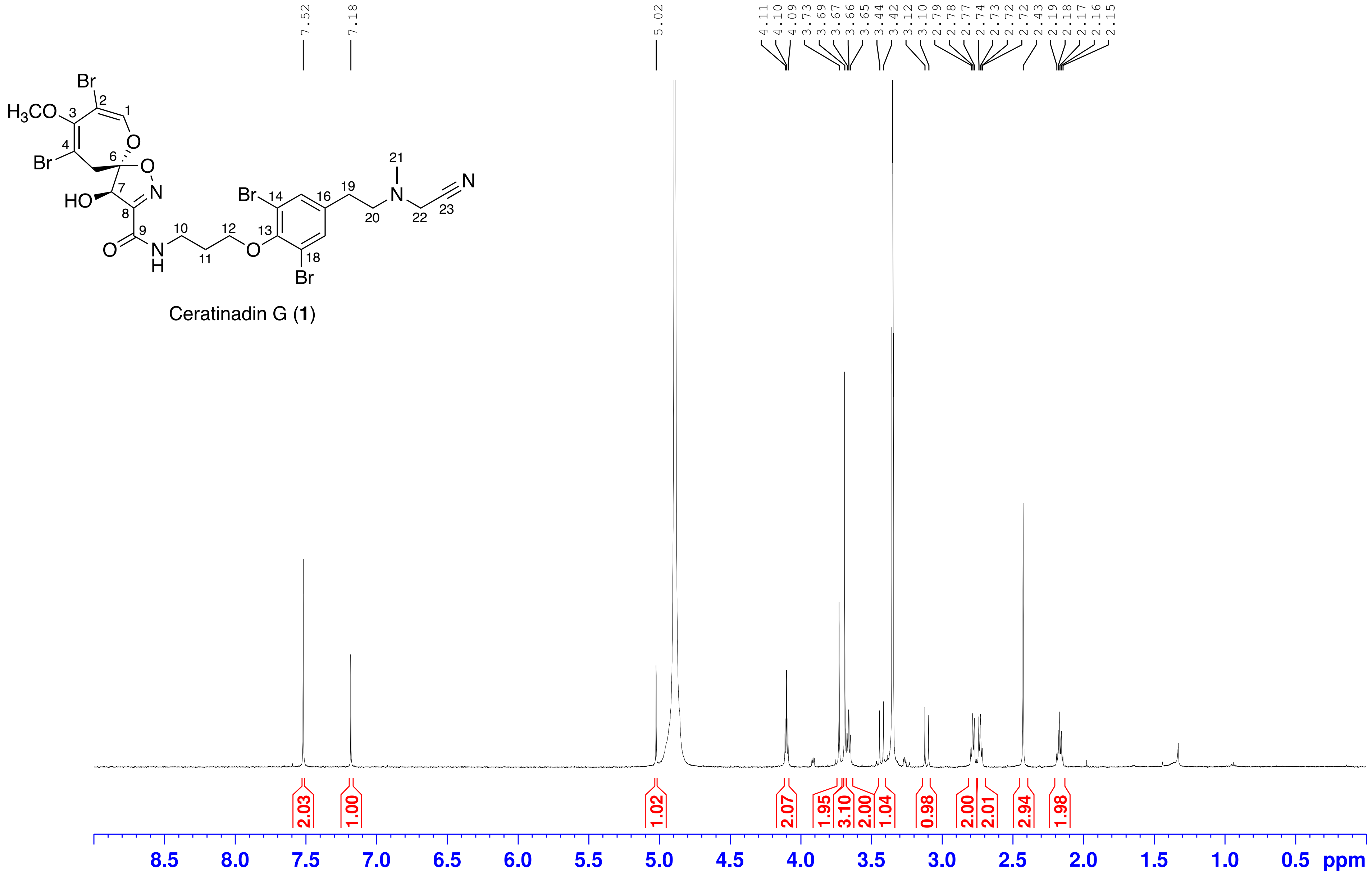

Figure S2: ^{13}C NMR spectrum of ceratinadin G (**1**) in CD_3OD (150 MHz).

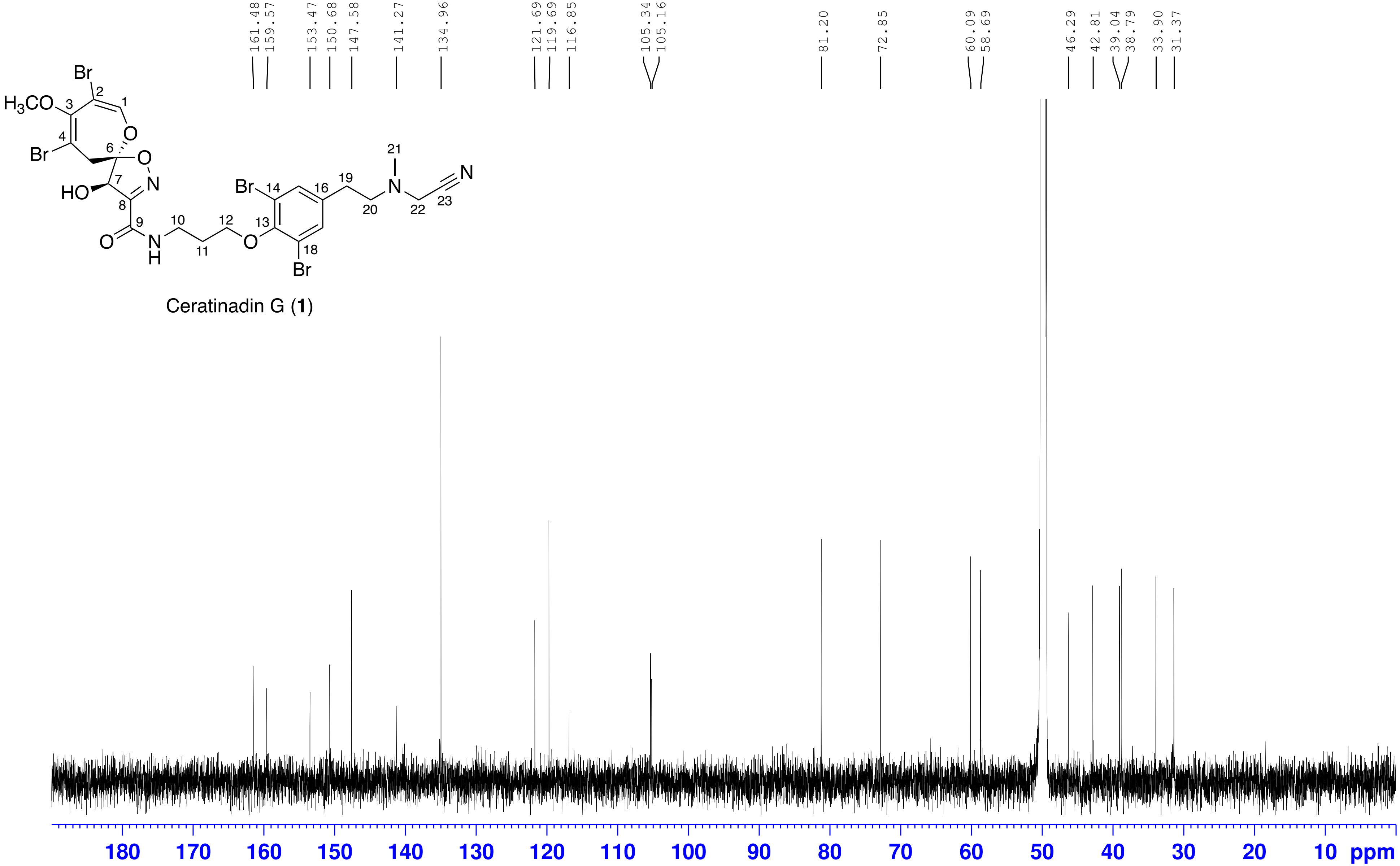

Ceratinadin G (**1**)

Figure S3: ^{1}H-^{1}H COSY spectrum of ceratinadin G (**1**) in CD_3OD (600 MHz).

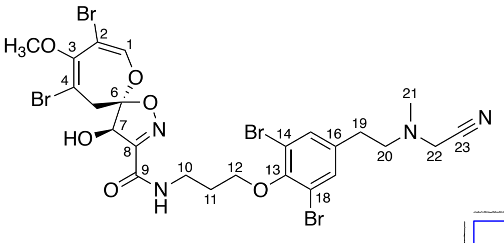

Ceratinadin G (**1**)

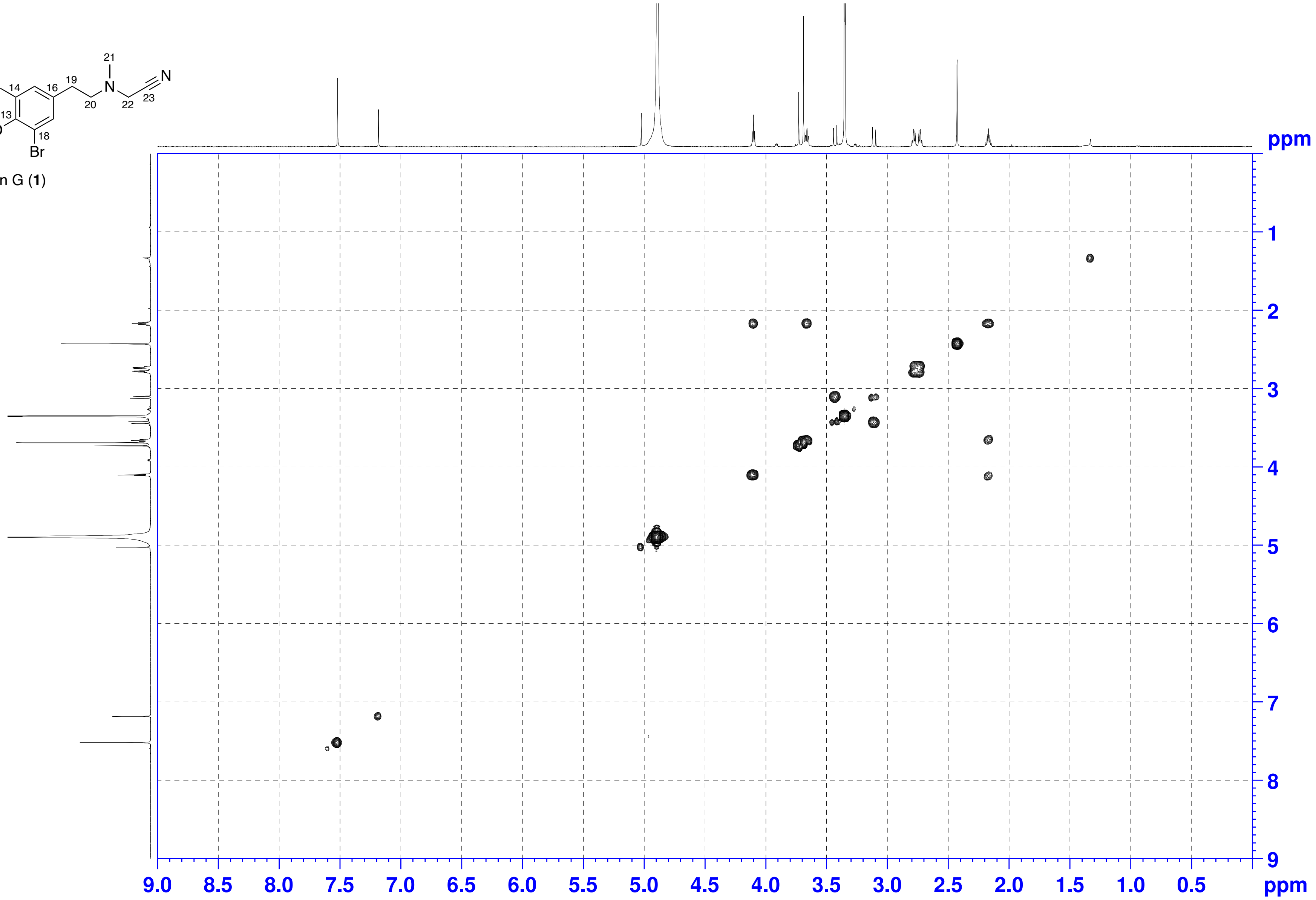

Figure S4: TOCSY spectrum of ceratinadin G (**1**) in CD_3OD (600 MHz).

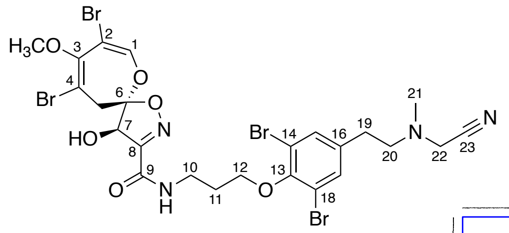

Ceratinadin G (**1**)

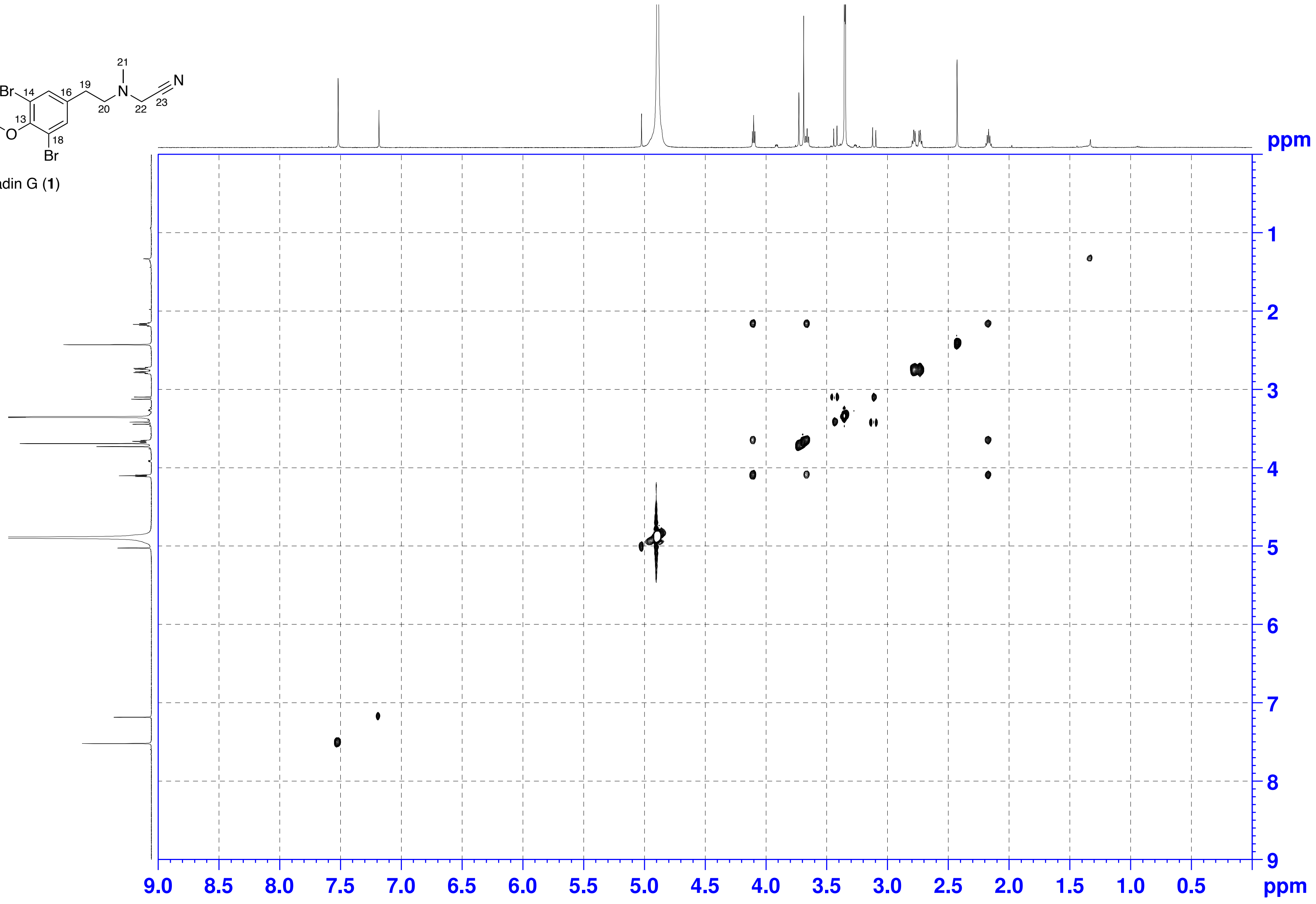

Figure S5: HSQC spectrum of ceratinadin G (**1**) in CD_3OD (600 MHz).

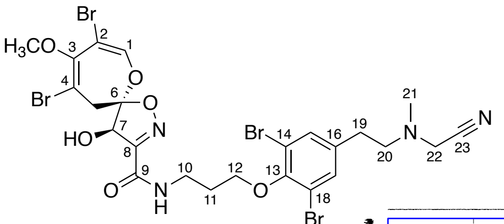

Ceratinadin G (**1**)

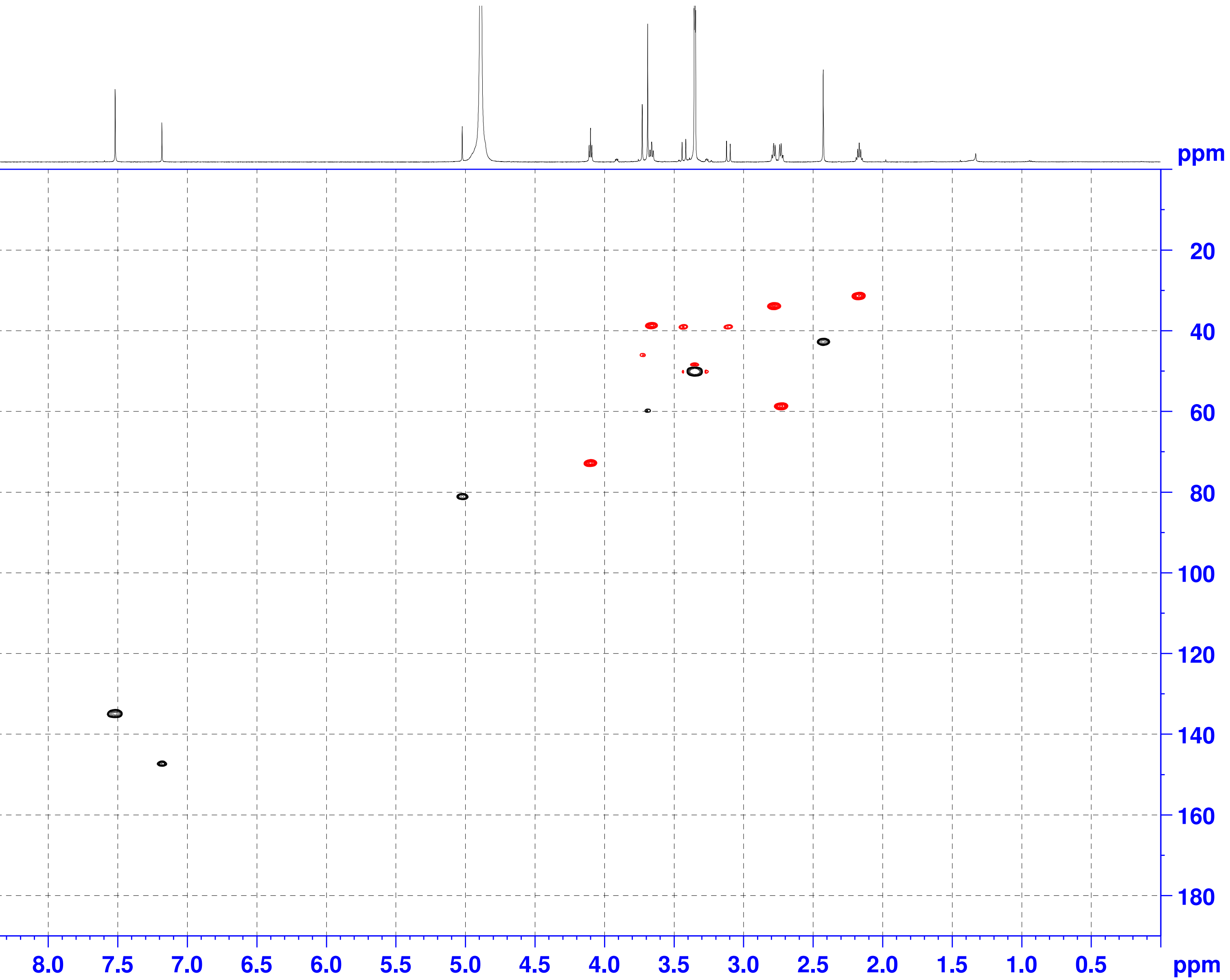

Figure S6: HMBC spectrum of ceratinadin G (**1**) in CD_3OD (600 MHz).

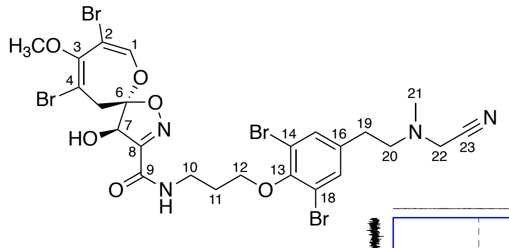

Ceratinadin G (**1**)

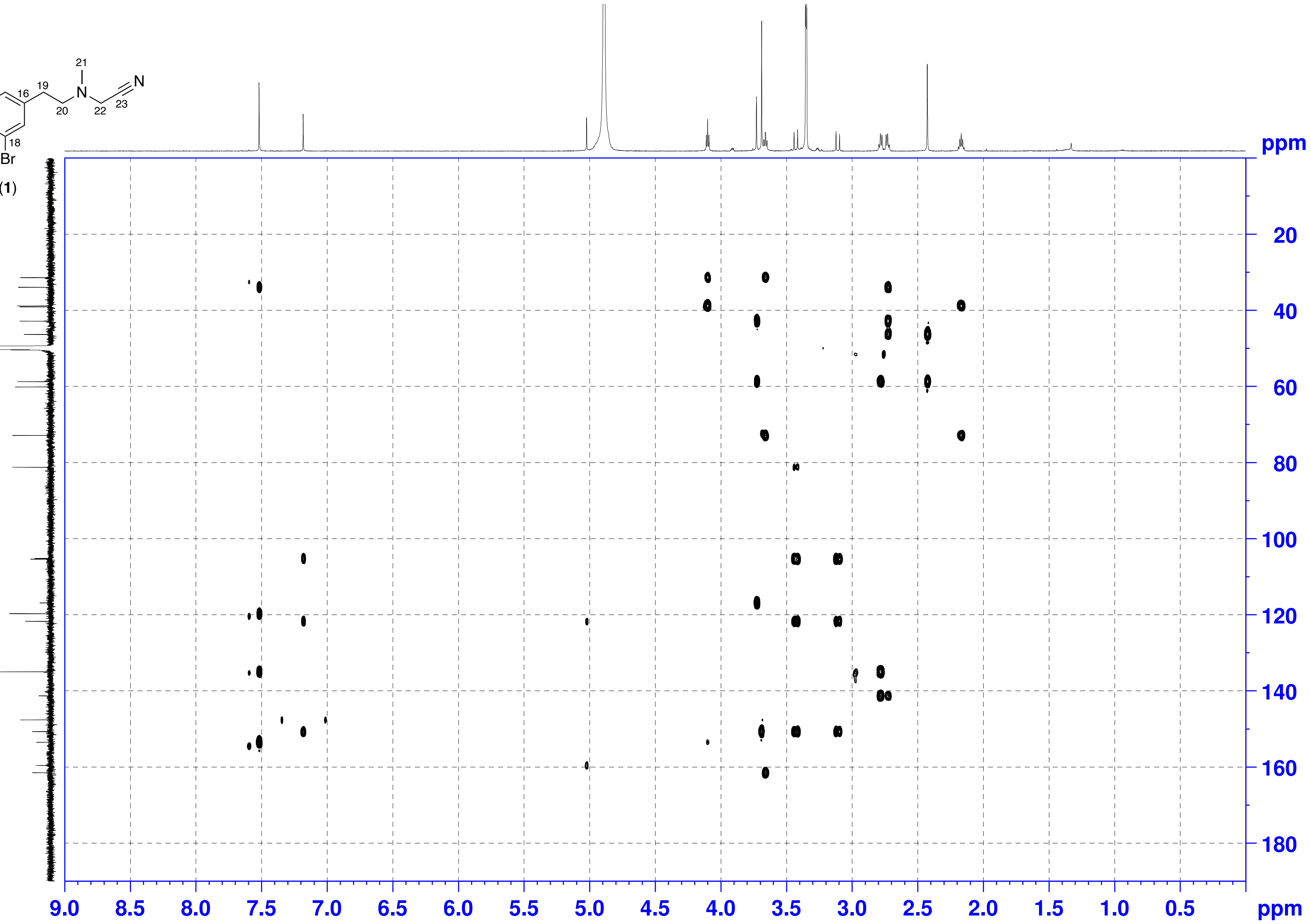

Figure S7: NOESY spectrum of ceratinadin G (**1**) in CD_3OD (600 MHz).

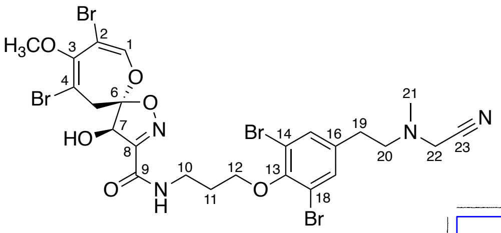

Ceratinadin G (**1**)

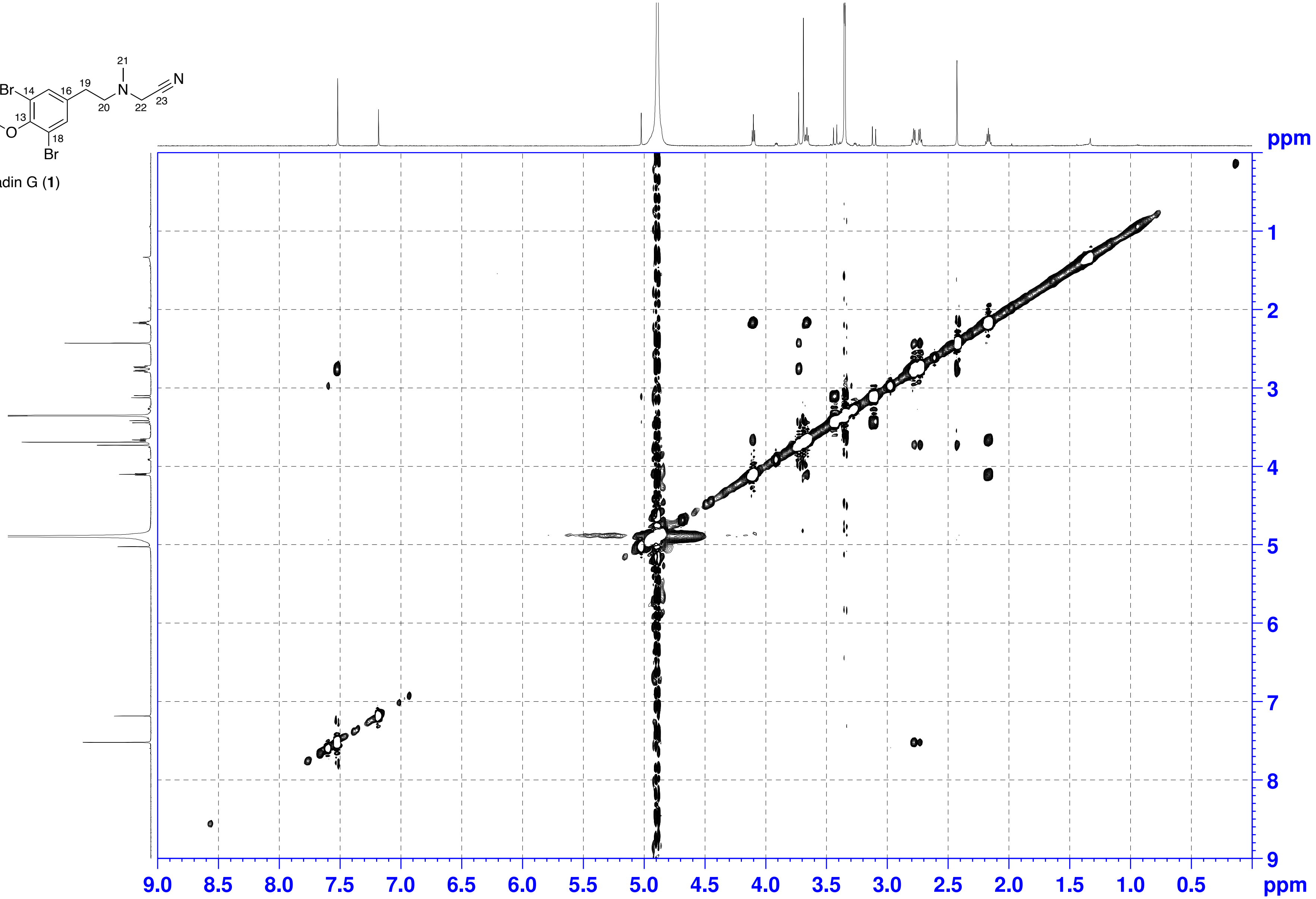

Figure S8: IR spectrum of ceratinadin G (**1**).

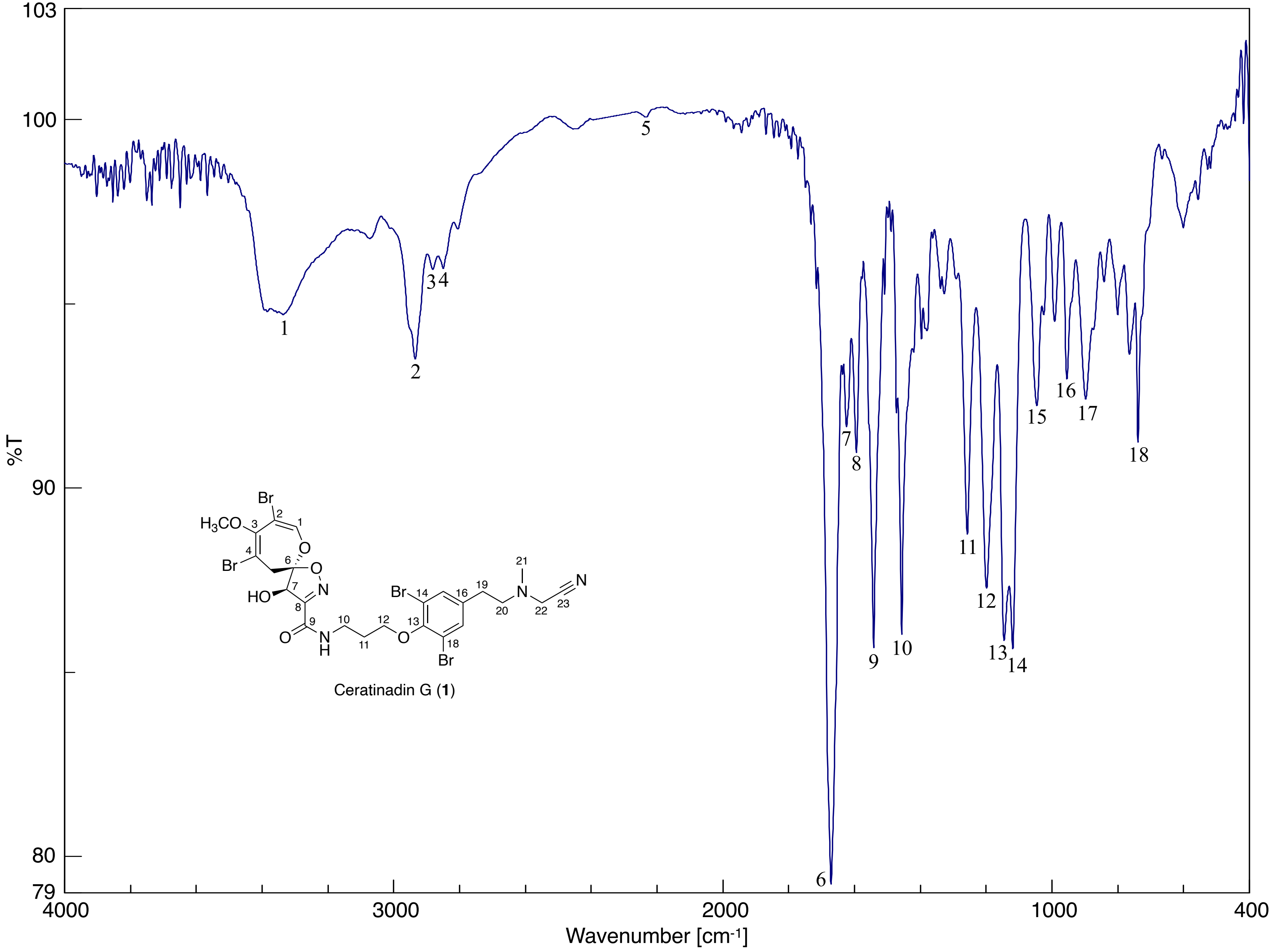

No.	Wavenumber (cm^{-1})
1	3337.21
2	2935.13
3	2878.24
4	2849.31
5	2234.13
6	1671.02
7	1623.77
8	1594.84
9	1541.81
10	1456.96
11	1257.36
12	1198.54
13	1144.55
14	1119.48
15	1046.19
16	953.627
17	897.701
18	737.639
